# Molecular analysis of exons 8, 9 and 10 of the fibroblast growth factor receptor 2 (**FGFR2**) gene in two families with index cases of Apert Syndrome

**Published:** 2015-09-30

**Authors:** Lilian Torres, Gualberto Hernández, Alejandro Barrera, Sandra Ospina, Rolando Prada

**Affiliations:** 1 Grupo Ciencias Básicas en Salud CBS. Fundación Universitaria de Ciencias de la Salud. Bogota, Colombia; 2 SaludCoop, Universidad del Rosario, Bogotá, Colombia; 3 Fundación Universitaria de Ciencias de la Salud. Hospital Infantil Universitario de San José. Bogotá, Colombia

**Keywords:** Apert syndrome, cleft palate, mutation, intron, *FGFR2* gene

## Abstract

**Introduction:**

Apert syndrome (AS) is a craniosynostosis condition caused by mutations in the Fibroblast Growth Factor Receptor 2 (*FGFR2*) gene. Clinical features include cutaneous and osseous symmetric syndactily in hands and feet, with variable presentations in bones, brain, skin and other internal organs.

**Methods:**

Members of two families with an index case of Apert Syndrome were assessed to describe relevant clinical features and molecular analysis (sequencing and amplification) of exons 8, 9 and 10 of *FGFR2* gen.

**Results:**

Family 1 consists of the mother, the index case and half -brother who has a cleft lip and palate. In this family we found a single *FGFR2* mutation, S252W, in the sequence of exon 8. Although mutations were not found in the study of the patient affected with cleft lip and palate, it is known that these diseases share signaling pathways, allowing suspected alterations in shared genes. In the patient of family 2, we found a sequence variant T78.501A located near the splicing site, which could interfere in this process, and consequently with the protein function.

## Abstract (es)

**Introducción:**

El síndrome Apert (SA) es un síndrome que cursa con craneosinostosis el cual es ocasionado por mutaciones en el gen del Receptor 2 del Factor de Crecimiento de Fibroblastos (*FGFR2*). Se caracteriza clínicamente por presentar sindactilias cutáneas y óseas en manos y pies de forma simétrica, cursa además con manifestaciones variables esqueléticas, cerebrales, en piel y otros órganos internos.

**Métodos:**

Miembros de dos familias con caso índice de Síndrome Apert fueron evaluados con el objetivo de describir las características clínicas relevantes y el análisis molecular (secuenciación y amplificación) de los exones 8, 9 y 10 del gen *FGFR2*.

**Resultados:**

La familia 1 está constituida por la madre, el caso índice y un medio hermano que presenta labio y paladar hendido. En esta familia solo se encontró la mutación S252W en la secuencia del exón 8 del gen *FGFR2* del caso índice. A pesar no encontrarse mutaciones dentro del estudio realizado al paciente afectado con labio y paladar hendido, se conoce que estas patologías comparten vías de señalización, lo que permite sospechar alteraciones en genes compartidos. En la familia 2, el resultado molecular del caso índice reportó la variante T78.501A en la secuencia del intrón 8, la cual se sitúa cercana al sitio de splicing, pudiendo alterar este proceso con una consecuente alteración de la función de la proteína.

## Introduction

Apert syndrome (AS) is an autosomal dominant disorder with a prevalence of 1-9/100,000 born, is characterized by synostosis of cranial sutures and acrocephaly, including brachycephaly, mid-facial hypoplasia, and syndactily of hands and feet 1.

During craniofacial development at different stages of embryonic formation, the signaling pathways of Fibroblast Growth Factors (FGF), their receptors (FGFR), and specifically the Fibroblast Growth Factor Receptor 2 (FGFR2) regulate the balance between proliferation and differentiation of progenitor osteogenic cells on the neural crest. Later in development these pathways are involved also in the formation of cartilage, skull bones and maxilla, as well as migration of the plates that give rise to the palate and lips 2. In 1995, Wilkie *et al*., demonstrated the presence of genetic mutations in the *FGFR2* gene in patients with AS, being P253R and S252W the most frequent mutations 3.

The *FGFR2* gene, located on chromosome 10, locus 10q26, encodes a transmembrane receptor with an extracellular region composed of three immunoglobulins-like domains IgI, IgII, and IgIII, a hydrophobic transmembrane segment, and a cytoplasmic tyrosine-kinase1 domain. Immunoglobulins domains correspond to the expression of exons 8, 9 and 10, where most patients (25 to 75%) have mutations 4.

Other mutations located in this region have been observed in Crouzon and Pfeiffer syndromes, without the appearance of craniosynostosis, and with the presence or absence of cleft lip and palate 5.

In this case report we want to expose two AS cases because of the importance it could have this information for the understanding of this pathology. The objective of family 1 is through molecular analysis identify if the index case and his brother with CLP diagnosed, have same mutation, there is considered a possible relationship between mutations at *FGFR2* and the phenotypes AS and CLP. The objective of family 2 is to identify the mutation.

## Materials and Methods

**Figure 1.**
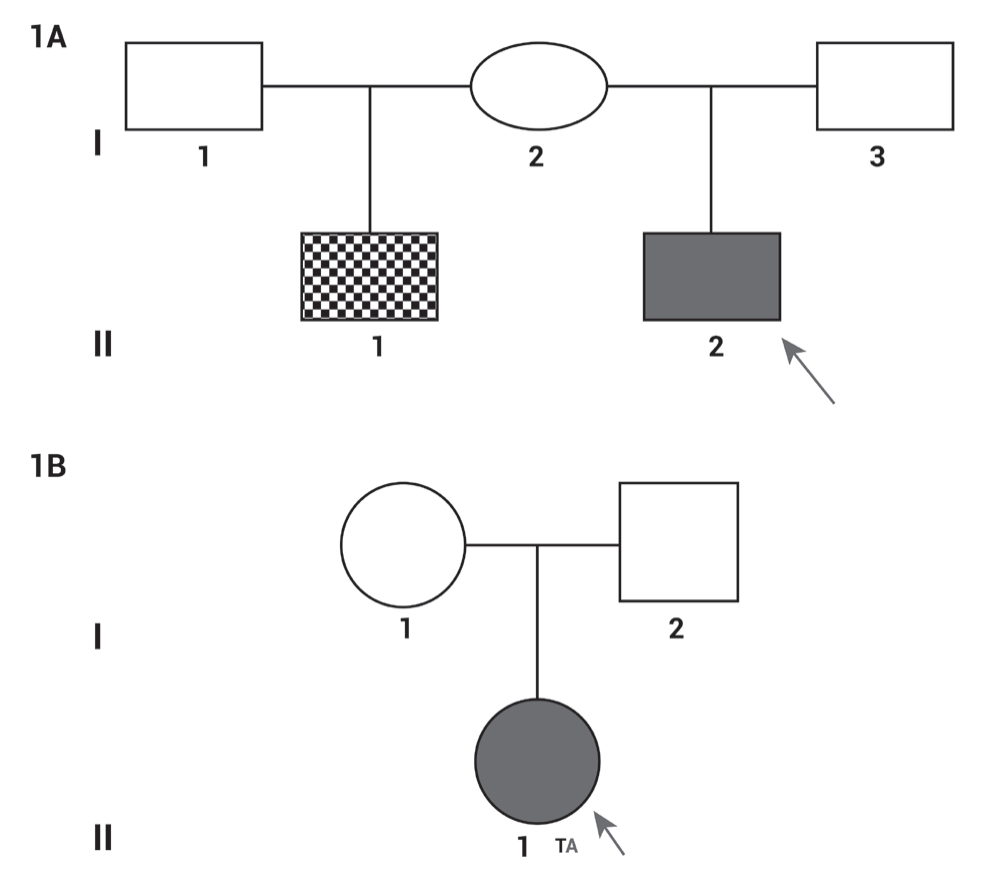
**A. **Genealogy Family 1. I, 2: Healthy mother. II, 1: Half-brother with CLP. II, 2: Index case of AS. **B**. Genealogy Family 2. I, 1 y I, 2: Healthy parents. II, 1: Index case of AS.

In the present case report we study two families with index case of AS, family 1: A healthy mother without features of craniosynostosis, the index case of AS and his half-brother with CLP diagnosed (I 1, II, 2 and II, 1) (Fig. 1A). Family 2: A healthy parents and the index case of AS (I 1, I 2 and II 1) (Fig. 1B).

Patients from two families diagnosed with AS were referred by the genetics and plastic surgery consultant to the genetics and molecular biology laboratory, where consent forms authorizing laboratory analysis and publication of genetic results were signed.

DNA from patient blood samples was isolated by the salting-out method, followed by PCR amplification of exons 8, 9 and 10 of the *FGFR2* gene. The primer sets for amplification of target exons were designed by using the NCBI reference sequence NW_004078068. Exon 8 (E8) amplification was performed using E8-forward primer 5`cccatgaaggagaccccagttg3´ and reverse primer 5`cattctcccatccccactccctc3´, for E9-forward 5´aatgctaagaccttcctggttgg3´ and reverse 5´cagtctcccaaagcaccaagtc3´, and for E10-forward 5ágccttctcagatggagccagg3´ and reverse primer 5´gagtctccatcctgggacatgg3´. The cycling conditions were as follows: 95° C for 3 min, followed by 35 cycles of 95° C for 1min, 58° C for 1 min, and a final step of 72° C for 1 min. Primers amplification specificity were tested by sequencing of PCR products, and sequenced data analyzed with BioEdit and Basic Local Alignment Search Tool (Blast) software. 

## Results

### Family 1

Consist in three persons, a healthy mother (I 1) and two children, the bigger one with CLP diagnosed (II 1) and the index case of AS (II 2) (Fig. 1A).

**Figure 2.**
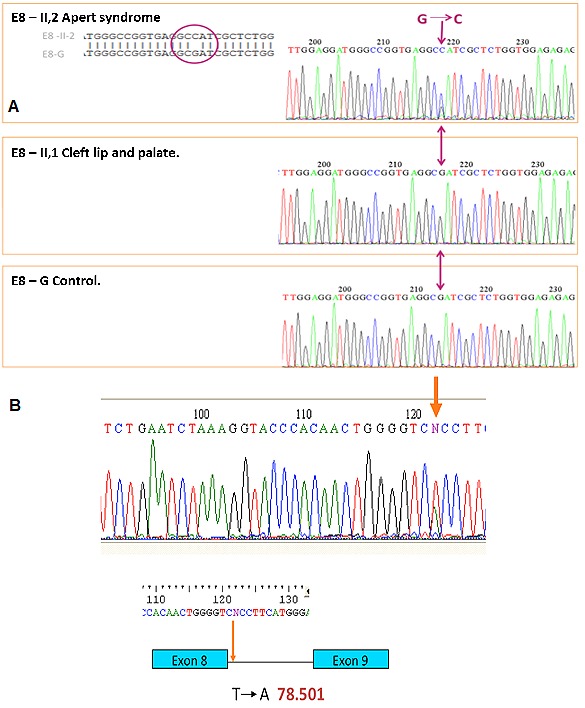
**A. **Family 1. I, 2: Healthy mother without mutation, II, 1: Male patient with cleft lip and palate defect (CLP) without mutation, and II, 2: Male patient with Apert syndrome having the S252W mutation in exon 8. **B. **Family 2. II, 1: male patient with Apert syndrome having a sequence variant T78.501A in intron 8.

**I, 2. **The mother of affected patients II, 1 and II, 2, without clinical features of craniosynostosis or CLP. Molecular analysis showed not changes in the reference sequences at exons 8, 9 and 10 (Fig. 2A).

**Figure 3.**
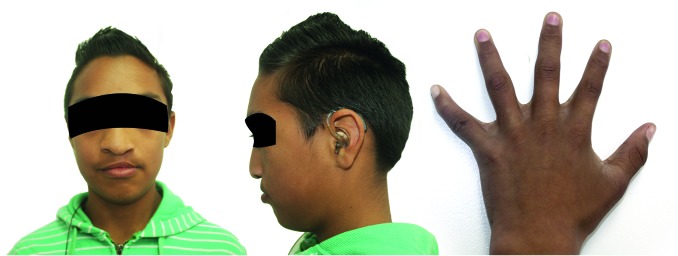
Male patient (II, 1) diagnosed with CLP, acrocephaly, aplasia cutis, hypertelorism, dysplastic ears, metaphyseal widening and vertebral involvement.

**II, 1. **The male patient was diagnosed with CLP defect, acrocephaly, aplasia cutis, hypertelorism, dysplastic ears, metaphyseal widening and vertebral involvement (Fig. 3). Clinical examination considered a possible craniosynostosis. However through molecular analysis, changes were no observed in sequences of exons 8, 9 and 10 (Fig. 2A). 

**Figure 4.**
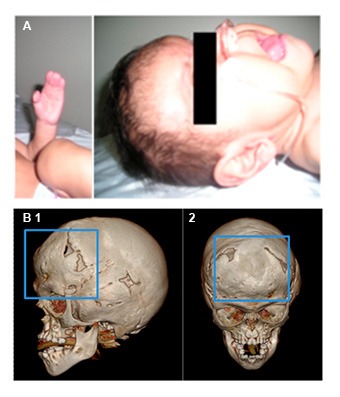
**A. **Male patient (II, 2) diagnosed with Apert Syndrome. Craniofacial features includes midface hypoplasia, low-set ears, depressed nose bridge, turricephaly, and osseous syndactyly in hands and feet. **B. **Skull computer tomography (CT) with three-dimensional (3D) model reconstruction of patient II, 2, Family 1. Several sutures synostosis (blue box) and midface hypoplasia shown in lateral (1) and frontal (2) images.

**II, 2.** A male patient who was the product of a second pregnancy. The patient's mother experienced preterm labor threat and received treatment for child lung maturation, and vaginal eutocic delivery at 37 weeks of gestation. Birth weight and length were 3,750 g and 50 cm, respectively. At one month of age the patient was diagnosed with AS and craniofacial features as midface hypoplasia, low-set ears, depressed nasal bridge, turricephaly, and digital syndactyly osseous in hands and feet (Fig. 4A), without family history or consanguinity. 

Imaging technology studies by transfontanelar ultrasonography and abdominal echography revealed a normal brain and internal organs. Similarly, a normal spine was observed by magnetic resonance images (MRI). However, restrictive inferior vena cava (IVC) was observed by echocardiogram. 

Comparative hand radiographs demonstrated the absence of the fourth digit, with fusion of the base of the fourth and fifth metacarpal. Thickening and shortening of the first, second, third and fifth digits, absence of middle phalanx in the second and third fingers, and arthrodesis in proximal and middle phalanx of the fifth finger in the right hand. The left hand had an apparent fusion of the third and fourth digits. There was no evidence of carpal commitment of the distal radius epiphysis and the ulna. Comparative feet radiographs showed a tendency to polydactyly.

Computed tomography of paranasal sinuses indicated underdevelopment of the maxillary sinuses, broad nasal bridge, hypertelorism, depth reduction of the optic orbit, with normal appearance for the optic nerves and muscles (Fig. 4B). Clinic history indicated that at 21 days of age the patient was hospitalized due to a transient tachypnea. Through the analysis of exon 8, 9 and 10 we could found an alteration in the sequence of exon 8, the mutation S252W, previously described in patients with AS (Fig. 2A). 

### Family 2

Consist in three persons, healthy parents (I 1 and II 2) and the index case of AS (II 1) (Fig. 1B).

**II, 1.** A patient who was diagnosed at birth with AS having turricephaly, brachycephaly, prominent forehead, midface hypoplasia, hypertelorism, depressed nasal bridge, malar hypoplasia and high palate. Both hands showed osseous syndactyly of digits 1 to 5, excluding the thumbs. Both feet had osseous syndactyly of all five digits without distal phalanx. Molecular analysis identified the variant T78.501A in the sequence, which is near to the hybridization site for one of the primers (Fig. 2B).

## Discussion

AS is a Syndromic Craniosynostosis, result of changes in DNA sequence, these mutations may happen on a genes list which involves the *FGF* and their receptors, especially *FGFR2*, being P253R and S252W the most frequent mutations. Since 1996 (Stanley et al) CLP was related to some patients with AS, especially the ones who present the S252W mutation. The relation of CLP with this mutation may be due to the shared pathways between these pathologies 5. In addition, the formation of nonsyndromic CLP, have been associated with the FGF and FGFR signaling pathways, where missense mutations D138N and R84S in *FGFR2* may alter protein function by changing the affinity of the receptor 6.

Therefore, it is possible that the origin of the phenotypic features in the two affected patients from Family 1 (bone involvement and craniofacial abnormalities), may be due to the same mutation in the *FGFR2* gene. Nonetheless, molecular analyses showed that patient II, 2 with AS has the S252W mutation. This mutation increases nonspecific receptor affinity to a subset of FGF, thereby allowing an inappropriate activation of the receptor by a gain of function, which would explain the patient phenotype 7. In patient II, 1 with CLP we did not found the same mutation, or another one in exons 8, 9 and 10, in which sequences were identical to those of his mother, who does not have the phenotypic characteristics of AS or CLP. These results clearly indicated that the phenotypic features of both affected patients may be of different origin.

This mutation increases nonspecific receptor affinity to a subset of FGF, thereby allowing an inappropriate activation of the receptor by a gain of function, which would explain the patient phenotype 7.

We could not rule out the origin of mutation in patient II, 1 with CLP features. However, isolated CLP, which is a complex congenital alteration with a multifactorial origin, in most cases, is caused by interaction between the environment and several genes involved with the FGF signaling pathway, including *FGFR1, FGFR 2 *and* FGFR 3, FGF2, FGF3, FGF4, FGF7, FGF8, FGF9, FGF10* and *FGF18, NUDT6, FCTBR*, and *PAX3* 8, among others which could be related to the this signaling pathway. Additional studies are required to determine whether mutations in any of these genes could be the origin of the patient phenotype.

From Family 2, we identified in patient II, 1 a DNA mutation near the flanking region of exon 8, 20bp from the intron start. Even though this type of variant has not been described in humans, studies in animal models have shown a gain of function of the *FGFR2* gene by modifying the intronic sequence that consequently alters the splicing site in exons. Therefore, the patient phenotype may be a result of the variance in the intron region of exon 8 that may affect the splicing and alter receptor function, as has been described in animal models.

Additional analysis of *FGFR2* mRNA or directly on the receptor may help to elucidate whether alteration of the splicing site may be influencing a gain of function 9. 

## Conclusions

AS and CLP are two different pathologies which share some pathways related with FGF and their receptors, among many others proteins, so it is possible that a change in the DNA sequence of genes involved may result in any of these phenotypes. However it is necessary to make extensive studies including more genes, allowing better view to determinate the presence or not of mutations.

Intronic mutations could have an effect on splicing site and then on the protein function, which could explain the phenotype of patient II, 1, at family 2. However to test this theory is necessary to carry out functional tests.

These findings are relevant because they complement the knowledge of the AS, allowing the establishment of new questions about the molecular bases and pathways associated with AS and associated pathologies as CLP.
